# Monensin and Nisin Affect Rumen Fermentation and Microbiota Differently *In Vitro*

**DOI:** 10.3389/fmicb.2017.01111

**Published:** 2017-06-16

**Authors:** Junshi Shen, Zhuang Liu, Zhongtang Yu, Weiyun Zhu

**Affiliations:** ^1^Laboratory of Gastrointestinal Microbiology, Jiangsu Key Laboratory of Gastrointestinal Nutrition and Animal Health, College of Animal Science and Technology, Nanjing Agricultural UniversityNanjing, China; ^2^Department of Animal Sciences, The Ohio State University, ColumbusOH, United States

**Keywords:** bacterial community, feed digestion, methane, microbial population, microbiota, monensin, nisin, rumen fermentation

## Abstract

Nisin, a bacteriocin, is a potential alternative to antibiotics to modulate rumen fermentation. However, little is known about its impacts on rumen microbes. This study evaluated the effects of nisin (1 and 5 μM) on *in vitro* rumen fermentation characteristics, microbiota, and select groups of rumen microbes in comparison with monensin (5 μM), one of the most commonly used ionophores in ruminants. Nisin had greater effects than monensin in inhibiting methane production and decreasing acetate/propionate ratio. Unlike monensin, nisin had no adverse effect on dry matter digestibility. Real-time PCR analysis showed that both monensin and nisin reduced the populations of total bacteria, fungi, and methanogens, while the population of protozoa was reduced only by monensin. Principal component analysis of bacterial 16S rRNA gene amplicons showed a clear separation between the microbiota shaped by monensin and by nisin. Comparative analysis also revealed a significant difference in relative abundance of some bacteria in different taxa between monensin and nisin. The different effects of monensin and nisin on microbial populations and bacterial communities are probably responsible for the discrepancy in their effects on rumen fermentation. Nisin may have advantages over monensin in modulating ruminal microbial ecology and reducing ruminant methane production without adversely affecting feed digestion, and thus it may be used as a potential alternative to monensin fed to ruminants.

## Introduction

The livestock industry nowadays faces three major challenges: feed shortage, environmental pollution, and food safety, for its sustainable development (Thornton, 2010). With ruminants, due to the rumen metabolism, approximately 2 to 12% of the feed energy is wasted as methane (Johnson and Johnson, 1995), which is 23 times more potent as a greenhouse gas than CO_2_ (FAO, 2006). Equally challenging if not more, much of the dietary nitrogen is converted to ammonia by rumen fermentation, and eventually more than 60% of the dietary nitrogen is excreted as urea via urine, which leads to serious groundwater pollution (Firkins et al., 2007). Various antimicrobials have been extensively used in animal production in the past 60 years to promote animal growth and health. Although antibiotics have been banned for non-therapeutic purposes in the European Union since the beginning of 2006 (Franz et al., 2010), monensin (**MON**) is still one of the most commonly used ionophores in ruminants in other countries. Benefits of feeding MON to ruminants include improved feed digestibility, a shift of volatile fatty acids (VFA) profiles toward increased propionate but decreased acetate and an associated decrease in methanogenesis, and decreased amino acid fermentation and ruminal ammonia concentration (Russell and Houlihan, 2003). However, MON lowers dry matter intake (DMI) and often suppresses milk fat in dairy cattle (Duffield et al., 2008), and its inhibitory effect on methane emissions can be transient (Johnson and Johnson, 1995; Guan et al., 2006). Moreover, the routine use of antibiotics in animal production has also been blamed for contributing to the emergence and widespread of antimicrobial resistance and occurrence of antibiotic residues in the environment and animal products (Molina et al., 2003). Thus, antibiotic use in animal production is restricted, and non-antibiotic alternatives are highly sought after to mitigate methane emission and nitrogen excretion (Jeyanathan et al., 2014; Patra and Yu, 2014).

Bacteriocins, which are low molecular weight antibacterial peptides, have been proposed as potential alternatives to antibiotics (Cotter et al., 2013; Paiva and Breukink, 2013). Nisin is a bacteriocin produced by certain strains of *Lactococcus lactis* ssp. *lactis*, and it has been widely used in the food industry to control pathogenic bacteria (Delves-Broughton et al., 1996). In recent years, nisin has also shown its potential in other fields, including biomedical application (Shin et al., 2016) and livestock husbandry (Jüzefiak et al., 2013). Several *in vitro* experiments using rumen microbiota have shown that nisin can suppress amino acid deamination and methanogenesis without negative impact on DM digestibility (Shen et al., 2016) or VFA production (Callaway et al., 1997; Sar et al., 2005). Some researchers cautioned that nisin could be susceptible to rumen proteases, which may limit its utilization *in vivo* (Russell and Mantovani, 2002). However, Lee et al. (2002) demonstrated that nisin binding to ruminal bacteria was faster than its degradation, and it can be used to modify ruminal fermentation. One *in vivo* study also showed a significant 10% decrease of methane emission in sheep supplemented with nisin (Santoso et al., 2004). Thus, it is important to understand the roles of nisin in modulating rumen microbiota and its fermentation and methanogenesis.

Both nisin and MON inhibit bacteria by primarily increasing the permeability of their cell membrane, but their mechanisms of inhibition are different. Nisin inhibits susceptible bacteria by creating pores that do not have selective permeabilities and by inhibiting cell wall synthesis (Cotter et al., 2013), while MON, which is an ionophore, functions as a Na^+^/H^+^ antiporter upon inserting itself into the cell membrane (Bergen and Bates, 1984). Therefore, we hypothesized that nisin and MON might impact the rumen microbiota composition differently, though they can achieve some similar effects on rumen function. To test this hypothesis, the present study, by integrating quantitative real-time PCR and Illumina sequencing of 16S rRNA gene amplicons, investigated the changes of rumen microbial composition and their fermentation profiles in response to MON and nisin using an *in vitro* model.

## Materials and Methods

### Experimental Design

Four treatments were used in this study. One treatment received sodium monensin (Solarbio, Beijing, China) at 5 μM (MON), which is equivalent to the widely used *in vivo* dose in ruminants (Callaway et al., 1997). Two treatments received nisin (1200 IU/mg of solid, about 3.0% of the solid; Jianglaibio, Shanghai, China) at 1 μM (NI1) and 5 μM (NI5). The negative control (NC) received neither additive. The doses of nisin were selected based on a previous study (Callaway et al., 1997). Each treatment had four replicates.

### Ruminal Inoculum and *In Vitro* Incubations

Three cannulated Holstein dairy cattle served as ruminal fluid donors for this *in vitro* study. The diet fed to these cattle contained (% DM basis) 20% corn silage, 40% grass hay (Chinese wild rye), and 40% concentrate mixture. The cows were fed twice daily at 06:00 and 18:00, and they had free access to feed and water. All animal protocols were approved by the Animal Care and Use Committee of Nanjing Agricultural University. Fresh ruminal fluid was collected through rumen cannula from the three cattle before morning feeding, mixed equally, and then poured into a sterilized bottle (1500 mL) leaving no headspace in the bottle, which was brought to the laboratory within 30 min. The mixed rumen sample was then squeezed through four layers of cheesecloth into a flask under CO_2_ in a water bath kept at 39°C until use.

The *in vitro* batch fermentation was carried out in 180 mL serum bottles. The fermentation substrate was a ground mixture of forage (25% corn silage, 17% alfalfa hay, and 8% Chinese wild rye) and concentrate (27% ground corn, 9% soybean meal, 6% cottonseed meal, 4% wheat bran, and 4% premix) at a 50:50 ratio. The buffered medium for the *in vitro* fermentation was prepared anaerobically as described by Theodorou et al. (1994). The anaerobic buffer medium and strained rumen fluid inoculum were combined in each bottle in a 9:1 (v/v) ratio under anaerobic conditions. A 100-mL mixture was immediately dispensed into each incubation bottle containing 1 g of ground feed substrate and respective additive. To prevent exposure to air, the headspace of the bottles was continuously flushed with CO_2_. Because monensin was not soluble in water, one concentrated stock solution (100×) was prepared using absolute ethanol. An equal volume of ethanol was also added to NC, NI1, and NI5. The final ethanol concentration was less than 1.0% (vol/vol). The serum bottles were each sealed with a butyl rubber stopper and secured with an aluminum crimp seal and incubated at 39°C for 24 h in a water bath with intermittent shaking by hand.

### Sampling and Chemical Analysis

Gas production was measured at 3, 6, 9, 12, and 24 h using a pressure transducer (Theodorou et al., 1994). After gas measurement at each time point, 30 μL of gas sample was drawn out immediately from each bottle using a gastight syringe to determine methane concentrations using gas chromatography (GC-2014, Shimadzu, Japan) (Yang et al., 2012). At the end of the 24 h of incubation, the pH value of each *in vitro* culture was measured immediately using a portable pH-meter (Ecoscan pH 5, Eutech Instruments, Singapore). Then, 1 mL of culture each was preserved by adding 0.2 mL of 25% HPO_3_ for VFA analysis using gas chromatography (7890A, Agilent, United Kingdom) according to the method described by Mao et al. (2008). Another 1 mL of each culture was collected for subsequent analysis for ammonia-N using a colorimetric method (Chaney and Marbach, 1962). Also, 1 mL of culture each was collected for DNA extraction and subsequent microbial analysis. All the samples were stored at –20°C until analyses. The remaining content of each culture was filtered through a filter bag (ANKOM Technology, United States) to analyze apparent dry matter digestibility (DMD) gravimetrically (Blümmel et al., 1997).

### DNA Extraction

Total metagenomic DNA was extracted using the bead-beating and phenol-chloroform extraction method as previously described (Dai et al., 2010). DNA was precipitated with ethanol and resuspended in 50 μL of Tris-HCl/EDTA buffer. The quality of the DNA extracts was visually checked using electrophoresis on 1.2% agarose gel (w/v) containing Goldview^TM^ (SaiBaiSheng, Shanghai, China), and the DNA concentration of each sample was determined using a Nanodrop 2000 (Thermo Fisher Scientific, Inc., Madison, WI, United States). The DNA samples were stored in –20°C until analyses.

### Quantitative Real-Time PCR Analysis

The PCR primers used for real-time PCR quantification of total bacteria, fungi, protozoa, methanogen, *Clostridium sticklandii*, and *Clostridium aminophilum* are listed in Supplementary Table S1. Among these microbial groups, *C. aminophilum* and *C. sticklandii* are two of the main hyper-ammonia-producing bacteria (**HAB**) isolated from the rumen contributing to elevated deamination therein (Russell et al., 1988). Plasmid DNA containing each cloned respective target sequence was obtained by PCR and cloning (Koike et al., 2007), and the resultant recombinant plasmids were used as the standard DNA in real-time PCR. Real-time PCR was performed on a StepOnePlus platform (Applied Biosystems, Foster City, CA, United States) using SYBR Premix Ex Taq dye (Takara). Quantification of copies of 16S rRNA gene (total bacteria, *C. sticklandii* and *C. aminophilum*), 18S rRNA gene (fungi and protozoa), and methyl coenzyme-M reductase gene (*mcr*A, for methanogens) in each sample was performed in triplicate, and the mean value was calculated. Standard curves were generated using 10-fold serial dilutions of each standard DNA containing the target gene sequences of the respective microbial group. The absolute abundance of each microbial population was expressed as copies of the target gene/mL of culture samples.

### Illumina Sequencing of 16S rRNA Gene Amplicons and Data Analysis

The V3-V4 hypervariable region of the 16S rRNA gene was amplified using primers 338 F (5′-ACTCCTACGGGAGGCAGCA-3′) and 806R (5′-GGACTACHVGGGTWTCTAAT-3′). Unique barcodes were added to the 5′ end of both primers for multiplexing. PCR products were examined on a 2% (w/v) agarose gel, and the expected bands were each extracted and purified using the AxyPrepDNA Gel Extraction Kit (Axygen Biosciences, Foster City, CA, United States). The concentrations of the purified DNA amplicons were each quantified using a QuantiFluor^®^ dsDNA kit (Promega, Madison, WI, United States). Amplicons from different samples were mixed in equal ratio and sequenced using the 2 × 300 pared-end kit on an Illumina MiSeq platform. The raw sequence reads were deposited into the NCBI Sequence Read Archive (SRA) database under the accession number SRP100539.

Raw fastq files were de-multiplexed, quality-filtered, and analyzed using QIIME 1.8.0 (Caporaso et al., 2010b) with the criteria as described by Mao et al. (2015b). Operational taxonomic units (OTUs) were *de novo* clustered with a 97% sequence similarity cutoff using UPARSE^1^ (version 7.1), and chimeric sequences were identified and removed using UCHIME (Edgar, 2010). The most abundant sequence within each OTU was designated as the ‘representative sequence’, and all the representative sequences were aligned against the core set of Greengenes 13.5 (DeSantis et al., 2006) using PYNAST (Caporaso et al., 2010a) with the default parameters set by QIIME. A PH Lane mask supplied by QIIME was used to remove the hypervariable regions from the aligned sequences. FASTTREE (Price et al., 2009) was used to create a phylogenetic tree of the representative sequences. Sequences were classified using the Ribosomal Database Project classifier with a standard minimum support threshold of 80% (Wang et al., 2007). Sequences identified as chloroplasts or mitochondria were removed before further analysis. Community alpha diversity measurements were estimated using the ACE, Chao1, Shannon, and Simpson indices. The interrelationships between the bacterial communities of different samples of the four treatments were visualized using principal component analysis (PCA), which was conducted using R to group the bacterial communities of different samples.

### Statistical Analysis

The real-time PCR data were log transformed to improve normality. All data (rumen fermentation characteristics, absolute abundance of the microbial groups quantified by qPCR, bacterial alpha diversity indices, and the relative abundances of microbial populations at the phylum, genus and OTU levels) were analyzed using the general linear model (GLM) procedure of SAS version 9.2 (SAS Institute Inc., Cary, NC, United States), and Duncan’s multiple comparison tests were used to assess differences between the means. Differences were considered statistically significant at *P* ≤ 0.05. Pearson correlation coefficients were calculated using SAS version 9.2 to examine the correlation between relative abundances of bacterial genera and each of the major fermentation data. Significant correlation was considered at *P* ≤ 0.05.

## Results

### Effects of Nisin and Monensin on Rumen Fermentation Characteristics

The fermentation characteristics of different treatments are summarized in **Table 1**. Both nisin and MON dramatically reduced gas and methane production compared with NC (*P* < 0.05), with MON resulting in the lowest gas production while NI5 leading to the lowest methane production. MON also significantly reduced DMD compared with NC (*P* < 0.05). However, nisin at either concentration had no adverse effect on DMD (*P* > 0.05), though NI5 had numerically lower DMD than NC. Nisin significantly increased ammonia concentration (*P* < 0.05) in a dose-dependent manner, whereas MON had no effect.

**Table 1:** Effects of nisin and monensin addition on gas and methane production, dry matter (DM) digestibility, and ammonia concentration in the *in vitro* rumen mixed cultures at 24 h.

Item	NC	MON	NI1	NI5	SEM	*P*-value
Total gas (mL)	181.9^a^	131.0^d^	162.2^b^	148.4^c^	1.48	<0.01
Methane (mL)	23.5^a^	14.5^b^	14.4^b^	10.6^c^	0.46	<0.01
DM digestibility (%)	58.4^a^	53.1^b^	59.1^a^	57.4^a^	0.53	<0.01
NH_3_-N (mM)	10.5^c^	10.7^bc^	11.2^b^	12.2^a^	0.19	<0.01
pH value	6.54^b^	6.60^a^	6.58^a^	6.59^a^	0.008	<0.01
Total VFA (mM)	80.3^a^	66.5^c^	69.3^b^	67.3^bc^	0.73	<0.01
Acetate (mM)	57.3^a^	43.2^b^	42.1^b^	36.6^c^	0.96	<0.01
Propionate (mM)	16.4^d^	20.9^c^	22.2^b^	26.2^a^	0.35	<0.01
Acetate/Propionate	3.50^a^	2.07^b^	1.90^b^	1.40^c^	0.086	<0.01
Butyrate (mM)	5.67^a^	2.05^d^	4.06^b^	3.51^c^	0.131	<0.01
Valerate (mM)	0.27^a^	0.08^c^	0.15^b^	0.14^b^	0.001	<0.01
Isobutyrate (mM)	0.29^a^	0.15^b^	0.31^a^	0.28^a^	0.011	<0.01
Isovalerate (mM)	0.34^c^	0.16^d^	0.41^b^	0.52^a^	0.016	<0.01
Total BCVFA (mM)	0.62^c^	0.30^d^	0.72^b^	0.81^a^	0.017	<0.01

*NC = negative control (no additives); MON = monensin, 5 μM; NI1 = nisin, 1 μM; NI5 = nisin, 5 μM.*

*^a-d^Means within a row with different superscripts differ (*P* < 0.05).*

Compared with NC, both MON and nisin significantly increased culture pH. Both MON and nisin reduced concentrations of total VFA, acetate, butyrate, valerate, and acetate/propionate ratio (*P* < 0.05), but increased propionate concentration, with MON resulting in a greater than nisin. The NI5 had the lowest acetate concentration and acetate/propionate ratio compared with other treatments. With branched-chain VFA (BCVFA), however, MON and nisin had different effects. Compared with NC, concentrations of isovalerate and total BCVFA were reduced by MON but were increased by nisin (*P* < 0.05). MON, but not nisin, decreased the concentration of isobutyrate (*P* < 0.05). Overall, the results indicate that MON and nisin showed different impacts on dry matter digestibility, ammonia production, and VFA profiles, especially BCVFA profiles.

### Effects of Nisin and Monensin on Rumen Microbial Populations

**FIGURE 1:**
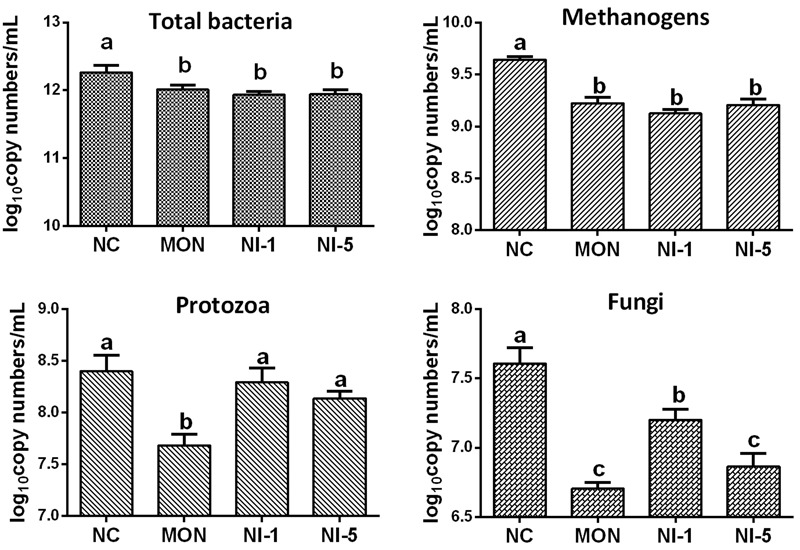
Effects of nisin and monensin addition on the population of total bacteria, methanogens, protozoa and fungi (log_10_ copy number of the target genes/mL) in the *in vitro* rumen mixed cultures. Values are means ± SE (*n* = 4). NC = negative control (no additives); MON = monensin, 5 μM; NI1 = nisin, 1 μM; NI5 = nisin, 5 μM. Bars with different letters (a, b, c) represent different means (*P* < 0.05).

**FIGURE 2:**
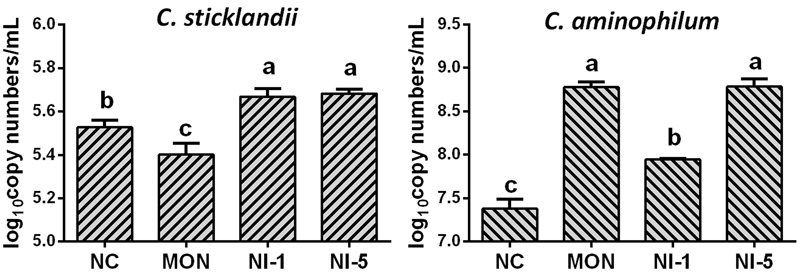
Effects of nisin and monensin addition on the population of *Clostridium sticklandii* and *C. aminophilum* (log_10_ copy number of 16S rRNA genes/mL) in the *in vitro* rumen mixed cultures. Values are means ± SE (*n* = 4). NC = negative control (no additives); MON = monensin, 5 μM; NI1 = nisin, 1 μM; NI5 = nisin, 5 μM. Bars with different letters (a, b, c) represent different means (*P* < 0.05).

Quantitative real-time PCR showed that both MON and nisin significantly reduced (*P* < 0.05) the population of total bacteria and methanogens compared with NC (**Figure 1**). Both MON and nisin also significantly reduced the population of fungi (*P* < 0.05), with the greatest reduction observed for MON followed by NI5 and NI1. Compared with NC, the population of protozoa was reduced by MON (*P* < 0.05) but not by nisin (*P* > 0.05). The HAB quantified, *C. aminophilum* and *C. sticklandii*, which have been shown previously to be sensitive to MON and nisin, responded differently to MON and nisin. Compared to that of NC, the population of *C. aminophilum* was increased (*P* < 0.05) by both MON and nisin (**Figure 2**), while that of *C. sticklandii* was decreased by MON but increased by nisin (*P* < 0.05). The results indicate that MON and nisin showed different effects on the populations of some major rumen microbes.

### Effects of Nisin and Monensin on Rumen Bacterial Communities

The effects of nisin and MON addition on the alpha diversity measurements of the ruminal bacterial community are summarized in **Table 2**. Across all 16 samples from the four treatments, 470,988 quality-checked sequences were classified as being bacterial. On average, at least 26,999 sequences per sample were obtained for all the treatments. The average length of the sequences was 448 bp. Greater than 99% depth coverage was achieved for all the samples. Both MON and nisin significantly reduced Shannon diversity index and increased the Simpson index compared with NC (*P* < 0.05), but the reduction or increase magnitudes were greater for MON than for nisin (*P* < 0.05). The numbers of OTUs were reduced by MON and NI5 (*P* < 0.05) but unaffected by NI1 (*P* > 0.05). The ACE and Chao 1 estimates of richness were not influenced by MON or nisin (*P* > 0.05).

**Table 2:** Effects of nisin and monensin addition on the alpha diversity measurements of ruminal bacteria at 3% dissimilarity level.

Item	NC	MON	NI1	NI5	SEM	*P*-value
# of sequences	26,999	29,585	29,205	31,958	2,255	0.51
Coverage (%)	99.6	99.6	99.6	99.5	0.04	0.57
OTU	665^a^	612^b^	644^ab^	613^b^	13.8	0.05
ACE	747	716	722	725	9.2	0.15
Chao 1	748	712	726	721	10.7	0.16
Shannon index	4.40^a^	3.71^d^	4.27^b^	4.03^c^	0.036	<0.01
Simpson index	0.039^c^	0.067^a^	0.041^c^	0.050^b^	0.002	<0.01

*NC = negative control (no additives); MON = monensin, 5 μM; NI1 = nisin, 1 μM; NI5 = nisin, 5 μM; OTU = operational taxonomic units; ACE = abundance-based coverage estimator.*

*^a-d^Means within a row with different superscripts differ (*P* < 0.05).*

**FIGURE 3:**
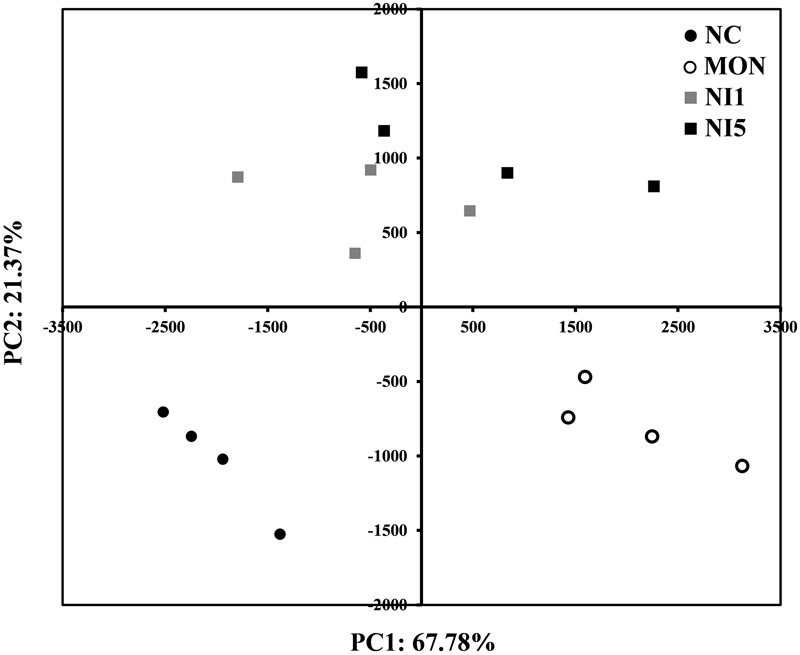
Principal component analysis (PCA) analysis of all samples based on the composition of bacterial communities. NC = negative control (no additives); MON = monensin, 5 μM; NI1 = nisin, 1 μM; NI5 = nisin, 5 μM.

In order to understand the impacts of MON and nisin on the overall rumen bacterial community, PCA analysis was performed (**Figure 3**). A clear separation was seen between NC and MON along PC1, which explains >67% of total variation, while NI1 and NI5 were separated from NC and MON along PC2, which explains >21% of total variation. The separation between NI1 and NI5 was minimal.

There were 13 bacteria phyla identified among all the treatments, with *Proteobacteria, Firmicutes*, and *Bacteroidetes* being the most predominant phyla, representing 39.3–56.8%, 25.9–30.2%, and 14.3–25.2% of all the sequences, respectively (**Table 3**). The relative abundance of the bacteria phyla was altered differently by the treatments (**Table 3**). Both MON and nisin decreased the relative abundance of *Firmicutes* compared with NC (*P* < 0.05), with MON corresponding to a greater decrease than nisin (*P* < 0.05). The relative abundance of *Bacteroidetes* and *Tenericutes* was significantly lower for MON and NI5 than for NC (*P* < 0.05), with the lowest observed for MON. On the contrary, the relative abundance of *Proteobacteria* was significantly higher for MON and NI5 than for NC (*P* < 0.05), with the highest observed for MON. The relative abundance of *Spirochaetae* was decreased by MON (*P* < 0.05) but not influenced by nisin (*P* > 0.05). The relative abundance of *Fibrobacteres* was decreased by MON and NI5 (*P* < 0.05) but increased by NI1 (*P* < 0.05).

**Table 3:** Effects of nisin and monensin addition on relative abundance of ruminal bacteria at phylum and genus level that each accounted for ≥0.5% of total sequences in at least one treatment.

Phylum	Genus/other	Relative abundance (%)				SEM	*P*-value
		NC	MON	NI1	NI5		
Proteobacteria	Total	39.3^c^	56.8^a^	41.1^c^	46.4^b^	0.84	<0.01
	***Ruminobacter***	30.2^b^	35.1^a^	25.6^c^	25.3^c^	0.83	<0.01
	*Succinivibrio*	6.02^d^	13.66^b^	11.38^c^	14.56^a^	0.248	<0.01
	*Succinimonas*	1.42^c^	1.95^a^	1.58^bc^	1.80^ab^	0.079	<0.01
	Unclassified *Succinivibrionaceae*	1.47^c^	5.49^a^	2.09^c^	4.18^b^	0.245	<0.01
Firmicutes	Total	30.2^a^	25.9^c^	27.0^c^	28.5^b^	0.41	<0.01
	***Pseudobutyrivibrio***	3.82^c^	1.30^d^	6.44^a^	4.98^b^	0.109	<0.01
	*Ruminococcus*	2.56^a^	0.54^b^	0.43^b^	0.38^b^	0.053	<0.01
	*Oribacterium*	2.81^c^	9.46^a^	5.78^b^	9.11^a^	0.220	<0.01
	***Butyrivibrio***	1.83^b^	0.79^d^	2.44^a^	1.35^c^	0.064	<0.01
	*Streptococcus*	1.61^a^	0.83^bc^	0.68^c^	0.75^b^	0.033	<0.01
	*Selenomonas*	1.14^c^	3.68^a^	2.45^b^	3.96^a^	0.126	<0.01
	*Roseburia*	0.69^b^	0.22^d^	1.00^a^	0.47^c^	0.027	<0.01
	*Anaerosporobacter*	0.71^a^	0.16^b^	0.17^b^	0.17^b^	0.021	<0.01
	***Succiniclasticum***	0.69^b^	1.84^a^	0.96^b^	1.05^b^	0.157	<0.01
	Unclassified *Lachnospiraceae*	7.40^a^	3.19^b^	3.08^b^	3.09^b^	0.063	<0.01
	Unclassified *Christensenellaceae*	4.08^a^	1.35^b^	1.53^b^	1.35^b^	0.099	<0.01
	Unclassified *Ruminococcaceae*	1.24^a^	0.58^c^	0.88^b^	0.56^c^	0.039	<0.01
**Bacteroidetes**	**Total**	25.2^a^	14.3^c^	27.0^a^	21.1^b^	0.68	<0.01
	*Prevotella*	16.30^a^	9.73^b^	17.30^a^	11.00^b^	0.806	<0.01
	***Bacteroides***	0.34^c^	0.99^a^	0.39^c^	0.63^b^	0.028	<0.01
	**Unclassified *Rikenellaceae***	5.93^b^	2.29^c^	6.40^ab^	7.04^a^	0.322	<0.01
	**Unclassified *Bacteroidales***	1.80^a^	0.61^b^	2.14^a^	1.73^a^	0.127	<0.01
	Unclassified *Prevotellaceae*	0.84	0.68	0.76	0.68	0.047	0.09
**Spirochaetae**	**Total**	2.41^a^	0.59^b^	1.75^a^	2.00^a^	0.210	<0.01
	***Treponema***	2.25^a^	0.58^c^	1.61^b^	1.93^ab^	0.192	<0.01
**Fibrobacteres**	**Total**	0.64^b^	0.07^d^	1.18^a^	0.43^c^	0.048	<0.01
	***Fibrobacter***	0.64^b^	0.07^d^	1.18^a^	0.43^c^	0.048	<0.01
Lentisphaerae	Total	0.78	0.79	0.57	0.64	0.097	0.34
	Unclassified *Lentisphaerae*	0.67	0.68	0.47	0.54	0.087	0.28
**Tenericutes**	**Total**	0.85^a^	0.13^c^	0.96^a^	0.40^b^	0.086	<0.01
	***Anaeroplasma***	0.76^a^	0.09^c^	0.80^a^	0.32^b^	0.068	<0.01

*NC = negative control (no additives); MON = monensin, 5 μM; NI1 = nisin, 1 μM; NI5 = nisin, 5 μM.*

*^a-d^Means within a row with different superscripts differ (*P* < 0.05).*

*The bacteria that were affected differently by monensin and nisin are bolded.*

A total of 69 genera of bacteria were identified, and these genera together accounted for 73.6–84.7 % of all the sequences. Only 17 of these genera were each represented by more than 0.50 % of the total sequences in at least one treatment (**Table 3**), and they were regarded as the “major genera”. Among the major genera, *Ruminobacter* was the most predominant accounting for 25.3–35.1 % of the total sequences, followed by *Prevotella* (9.73–17.30 %), *Succinivibrio* (6.02–14.56 %), and *Pseudobutyrivibrio* (1.30–6.44 %). In addition, a large portion (15.3–26.4 %) of the sequences could not be classified to a known genus. Unclassified *Lachnospiraceae* (3.08–7.40 %) and unclassified *Rikenellaceae* (2.29–7.04 %) were the first and the second most predominant unclassified groups, respectively. At the genus level, MON and nisin showed different effects on some bacterial genera. Compared with NC, the relative abundance of *Ruminobacter* was increased by MON but decreased by nisin irrespective of concentration (*P* < 0.05). On the contrary, the relative abundance of *Pseudobutyrivibrio* and unclassified *Rikenellaceae* was decreased by MON but increased by nisin at both concentrations (*P* < 0.05). The relative abundance of unclassified *Bacteroidales* was decreased by MON (*P* < 0.05) but not influenced by nisin (*P* > 0.05). The relative abundance of *Butyrivibrio, Roseburia*, and *Fibrobacter* was decreased by both MON and NI5 but increased by NI1 (*P* < 0.05). In contrast, the relative abundance of *Prevotella* and *Anaeroplasma* was decreased by MON and NI5 (*P* < 0.05) but not influenced by NI1 (*P* > 0.05). Besides, MON and nisin also had a parallel influence on some bacterial genera. The relative abundance of *Succinivibrio, Succinimonas, Oribacterium, Selenomonas*, and unclassified *Succinivibrionaceae* was increased, whereas that of *Ruminococcus, Streptococcus, Anaerospoobacter, Treponema*, unclassified *Lachnospiraceae*, unclassified *Christensenellaceae*, and unclassified *Ruminococcaceae* was decreased by MON and nisin at both concentrations (*P* < 0.05).

A total of 838 OTUs were clustered at a 0.03 dissimilarity cut-off across all the samples, and 46 OTUs were represented by more than 0.50 % of the total sequences in at least one treatment (Supplementary Table S2). Among these, eight OTUs were decreased (*P* < 0.05), six OTUs were increased (*P* < 0.05), and two OTUs were not influenced (*P* > 0.05) by MON and nisin at both concentrations. However, MON and nisin had different effects on the relative abundance of most OTUs. Compared with NC, four OTUs were decreased by MON (*P* < 0.05) but increased by nisin irrespective of concentration (*P* < 0.05). Five OTUs were increased by MON (*P* < 0.05) but decreased (*P* < 0.05) or unchanged (*P* > 0.05) by nisin at both concentrations. One OTU was unaffected by MON (*P* > 0.05) but decreased by NI1 and NI5 (*P* < 0.05). Furthermore, nisin also showed significant dosage effect on 20 OTUs compared with MON.

### Correlations Between the Relative Abundance of Rumen Bacteria and Fermentation Parameters

**FIGURE 4:**
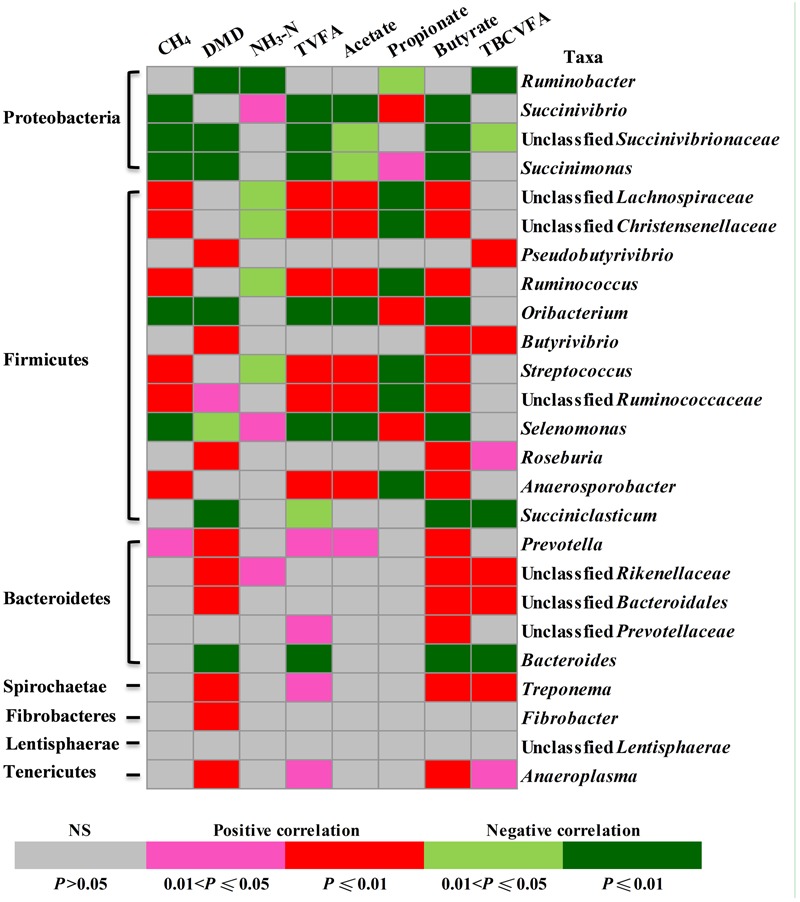
Correlations between the relative abundance of rumen bacteria (at genus level) and fermentation parameters. Cells are colored based on Pearson correlation coefficient.

The relative abundance of some of the identified rumen bacterial genera or equivalent taxa (referred to as genera) appeared to be correlated with fermentation characteristics (**Figure 4**). Seven genera were positively and five taxa were negatively correlated with methane production; 11 taxa were positively and seven taxa were negatively correlated with DMD; three taxa were positively and five taxa were negatively correlated with ammonia concentrations; 10 taxa were positively and seven taxa were negatively correlated with total VFA concentrations; seven taxa were positively and five taxa were negatively correlated with acetate concentrations; four taxa were positively and seven taxa were negatively correlated with propionate concentrations; 14 taxa were positively and seven taxa were negatively correlated with butyrate concentrations; and seven taxa were positively and four taxa were negatively correlated with total BCVFA concentration.

## Discussion

Microbes are solely responsible for feed digestion and the production of methane, VFA, and ammonia in the rumen (Patra and Yu, 2014). Previous studies have demonstrated that MON, an ionophore, and nisin, a bacteriocin, could achieve similar impacts on methane and VFA production (Callaway et al., 1997; Shen et al., 2016) but showed different effects on feed digestion (Shen et al., 2016). Therefore, comprehensive characterization of microbial populations and communities are essential to understand the mode of effects of nisin and monensin on feed digestion and rumen fermentation. This study, combining high-throughput sequencing and quantitative real-time PCR using an *in vitro* fermentation system, for the first time revealed the different effects on bacterial groups. Moreover, this study also shines new light on the potency of nisin as an alternative to monensin in modulating rumen fermentation.

### Effects of Nisin and MON on the Major Microbial Groups and Bacterial Community Involved in Feed Digestion

As expected, monensin decreased feed digestion, consistent with previous studies (Russell and Strobel, 1988; Narvaez et al., 2013). Cellulolytic bacteria in the rumen are the major contributors to fiber degradation (Jeyanathan et al., 2014). The adverse effect of monensin on feed digestion is mainly attributed to its inhibition of cellulolytic bacteria (Russell and Strobel, 1989; Narvaez et al., 2013). *F. Succinogenes, R. flavefaciens* and *R. albus* are considered the major cellulolytic bacterial species cultured because of their high cellulose digestion ability (Krause et al., 2003). *Butyrivibrio, Pseudobutyrivibrio, Oscillibacter*, and *Eubacterium* are also recognized as fibrolytic bacterial genera (Thoetkiattikul et al., 2013). However, a meta-analysis by Koike and Kobayashi (2009) revealed that the so far recognized fibrolytic species might represent only a small proportion of the total fibrolytic population in the rumen. Indeed, a recent study reported that high abundance of some unclassified groups, including those assigned to *Lachnospiraceae, Christensenellaceae, Ruminococcaceae, Rikenellaceae, Prevotellaceae*, and *Bacteroidales*, have been found tightly adhering to forages after incubation in the rumen, indicating that these new taxa may play an important role in forage degradation in the rumen (Liu et al., 2016). In the present study, the reduced abundance of fibrolytic bacterial genera (e.g., *Ruminococcus, Butyrivibrio, Pseudobutyrivibrio*, and *Fibrobacter*) and potential fibrolytic bacteria taxa (e.g., unclassified *Lachnospiraceae*, unclassified *Christensenellaceae*, unclassified *Ruminococcaceae*, unclassified *Rikenellaceae*, and unclassified *Bacteroidales*) might have resulted in the decreased feed digestion in the monensin treatment. In addition, fungi and protozoa are known to contribute to fiber degradation (Martinez-Fernandez et al., 2016). Hence, the reduced fungi and protozoa populations in the MON treatment may be another reason for the decreased feed digestion therein.

The monensin addition decreased feed digestion with a concomitant reduction in methane production, which is consistent with the notion that inhibition of methanogenesis generally results in decreased feed digestion and fermentation in batch cultures (Ungerfeld, 2015). However, unaffected fiber digestibility coupled with reduced methane production in several *in vitro* experiments has also been reported (Morgavi et al., 2010), and the authors believed that the increased *Fibrobacter* population observed in these studies might have compensated fiber degradation. This phenomenon has also been found in the nisin treatments in the present study, in which methane was decreased greatly, while feed digestion was unaffected. This may be explained by shifts in microbial populations. In the nisin treatments, the relative abundance of some Gram-positive fibrolytic bacterial genera, such as *Ruminococcus*, unclassified *Lachnospiraceae*, unclassified *Christensenellaceae*, and unclassified *Ruminococcaceae* was decreased. However, besides *Fibrobacter* as mentioned by Morgavi et al. (2010), nisin treatment increased or maintained high relative abundance of some other fibrolytic bacterial genera, such as *Pseudobutyrivibrio, Butyrivibrio*, unclassified *Rikenellaceae*, and unclassified *Bacteroidales*, all of which were decreased by MON. These functionally not yet well recognized minor groups may also be important fibrolytic bacteria and contribute to the different impacts between nisin and MON. Moreover, the population of protozoa was also unaffected by nisin. Therefore, the increase or maintenance of these fibrolytic microbes in the nisin treatments might have allowed persistent efficient fiber degradation. These data suggested that the different effects of monensin and nisin on microbial community, particularly fibrolytic bacterial composition might have resulted in their discrepancy on feed digestion.

### Effects of Nisin and MON on Methane Production and Related Changes in Major Microbial Groups and Bacterial Community

Corroborating the findings of several previous studies (Callaway et al., 1997; Shen et al., 2016), the present study also showed reduced methane production by nisin and monensin, with reduction by 38.7%, 54.9%, and 38.1% in the NI1, NI5, and MON treatments, respectively. However, the mechanism of action on methane production is probably different between MON and nisin. In the present study, both MON and nisin significantly reduced the population of methanogens. Narvaez et al. (2013) also found reduced methanogens population with monensin addition. However, it has been demonstrated that monensin does not directly inhibit methanogen, and it can directly inhibit H_2_-producing bacteria, thereby indirectly decreasing methane production (Russell and Houlihan, 2003). Supporting this notion, the present study also showed a decrease in H_2_-producing microorganisms, including protozoa, fungi, and Gram-positive taxa of *Firmicutes*, to which the primary ruminal H_2_-producing bacteria belong. Different with our *in vitro* result, one recently published *in vivo* study showed no effect of monensin on archaea population (Schären et al., 2017). Undoubtedly, their results further confirmed that the inhibition of methanogenesis by monensin is most likely caused by a decrease in substrate availability, rather than by direct inhibition to methanogens. In contrast, nisin has no influence on protozoa, which play an important role contributing to methane production (Morgavi et al., 2010). It should be noted that nisin also inhibited fungi and some Gram-positive taxa of *Firmicutes*, but to a less extent than monensin. Hence, the observed decrease in methane production by nisin is probably partly due to indirect inhibition to H_2_-producers. Besides, as proposed but not substantiated by Santoso et al. (2004), nisin probably reduced methane production by direct inhibition to methanogens. However, this premise can not be confirmed directly by the present study either. Therefore, future studies using pure cultures of methanogens are warranted to directly and definitively approve if nisin can directly inhibit methane production.

### Effects of Nisin and MON on VFA Production and Related Changes in Rumen Microbial Composition

In the present study, both monensin and nisin additions caused an increase of propionate concentration but a decrease of acetate concentration, resulting in a decrease in the ratio of acetate to propionate. These results are consistent with those of previous *in vitro* studies (Callaway et al., 1997; Shen et al., 2016). In the rumen, propionate is produced through two pathways: the succinate pathway and the acrylate pathway, and the succinate pathway is the major pathway (Jeyanathan et al., 2014). Succinate is formed as an intermediate but not as an end product of the rumen fermentation (Scheifinger and Wolin, 1973). *Fibrobacter succinogenes* (Jeyanathan et al., 2014) and members of the *Succinivibrio* (Pope et al., 2011) produce succinate as their principal fermentation end product, while members of *Selenomonas* (e.g., *Selenomonas ruminantium*; Scheifinger and Wolin, 1973) produce propionate via the succinate pathway. In the present study, the abundance of *Fibrobacter* was much less than that of *Succininivibrio*. Therefore, the greatly increased relative abundance of *Succinivibrio* (>11.38%) and *Selenomonas* (>2.45%) in the MON and the nisin treatments probably contributed to increased propionate via the succinate pathway. Similar to our results, an *in vivo* study also found an increase in ruminal propionate proportions after monensin addition, which was caused by an increase in abundance of succinate and propionate producers and a decrease in non-producers (Schären et al., 2017). In addition, the acrylate pathway is also an important propionate-producing pathway in the rumen, in which lactate-producing bacteria such as *Streptococcus bovis* play a key regulatory role (Jeyanathan et al., 2014). However, the relative abundance of *Streptococcus* in the MON and the nisin treatments was reduced significantly. Therefore, propionate production through the acrylate pathway was probably weakened by both MON and nisin. These results suggest that monensin and nisin may increase propionate production through the succinate pathway.

The reduced acetate production in the MON and the nisin treatments can be explained by the decrease of some Gram-positive fibrolytic bacteria, such as *Ruminococcus* spp., which are major acetate-producing bacteria (Jeyanathan et al., 2014). In addition, the present study also revealed that some unclassified Gram-positive bacteria, such as unclassified bacteria in *Lachnospiraceae, Christensenellaceae*, and *Ruminococcaceae*, were positively correlated with acetate concentration. Thus, decreased populations of these three unclassified groups might have also contributed to the reduction in acetate production in the MON and the nisin treatments. However, nisin increased propionate production and reduced acetate production to a greater magnitude than monensin. Nisin also increased butyrate concentration to a greater extent than monensin. Protozoa were among the major butyrate producers in the rumen (Williams and Coleman, 1997), and they were positively associated with butyrate production (Mao et al., 2015a). In the present study, protozoa were decreased by monensin but not affected by nisin. Hence, the different effect on protozoal population may partly explain the higher butyrate concentration in the nisin treatment than in the MON treatment. This study observed that the relative abundance of *Butyrivibrio* and *Pseudobutyrivibrio* in the nisin treatments was significantly higher than in the MON treatment. *B. fibrisolvens* (Stewart et al., 1997) and *Pseudobutyrivibrio xylanivorans* (Kopecny et al., 2003) are important butyrate-producing species in the rumen. Therefore, the higher relative abundance of butyrate-producing bacteria in the nisin treatments may further explain the higher butyrate concentration therein than in MON treatment.

### Effects of Nisin and MON on Ammonia Production and Related Changes in Some Important Microbial Groups

Ammonia concentration in ruminal *in vitro* batch cultures only depends on the balance between the rate of formation and utilization of ammonia by microbes. In the present study, microbial crude protein (MCP) was not measured, but the decreased bacterial populations in the nisin and the MON treatments suggested reduced ammonia utilization by microbes. Therefore, increased ammonia concentration should be expected if the formation rate was not decreased. However, the MON treatment had similar ammonia concentration as the control. It is speculative, but this may result from reduced amino acid deamination by microbes, which has been reported in earlier studies (Yang and Russell, 1993; Callaway et al., 1997). This is substantiated by the lower concentrations of BCVFA and valerate, both of which primarily arise from deamination of amino acids (Patra and Yu, 2014). Besides, we also quantified the population of protozoa, which are known protein degraders and net ammonia-producer (Firkins et al., 2007), and that of *C. aminophilum* and *C. sticklandii*, both of which are **HAB** (Russell et al., 1988). The reduced population of protozoa and *C. sticklandii* by monensin corroborates the reduced total amino acid deamination further. However, *C. aminophilum* increased in response to monensin, a finding contradictory to a report by Callaway et al. (1997), who found that *C. aminophilum* in pure cultures was inhibited by monensin though it has greater resistance than *C. sticklandii*. In contrast, the nisin treatments had similar total BCVFA and valerate concentrations as the control, suggesting enhanced amino acid deamination per unit of bacterial biomass. This was substantiated by the increased *C. sticklandii* and *C. aminophilum* populations, though protozoa were unaffected. Hence, the reduced ammonia utilization and enhanced amino acid deamination per unit of bacterial biomass might have contributed to the increased ammonia concentration in the nisin treatments. In an *in vitro* study, Sar et al. (2005) also found increased ammonia concentration after nisin addition. Contradictory to our results, Callaway et al. (1997) reported that nisin inhibited amino acid degradation by the above two HAB, and nisin was more effective than monensin in inhibiting the growth of *C. aminophilum*. The discrepancy in the effects of monensin and nisin on HAB populations between pure cultures and mixed cultures cannot be easily explained, but these (and possibly other) HAB may respond to them differently in mixed cultures than in pure cultures.

### Alteration in Rumen Microbial Fermentation Profiles Correlated with Changes in Bacterial Community

Feed degradability, methane production, VFA profiles, and ammonia production in the rumen are some of the most important parameters indicative of microbial metabolism therein (Patra and Yu, 2015). To explore the correlations between rumen bacterial shifts (at genus level) and fermentation characteristics, Pearson’s correlation analysis was performed. This study observed a large portion of unclassified bacteria, including those unclassified within *Lachnospiraceae, Rikenellaceae, Christensenellaceae, Ruminococcaceae*, and *Bacteroidales*, which have not yet been functionally characterized. The correlation analysis revealed that the dynamic changes of these unclassified groups in response to nisin and monensin are probably responsible for or caused by, at least partially, the shifts in these fermentation characteristics. However, correlation is not causation, and a particular taxon found in association with a parameter may be merely a bystander (Hanage, 2014). It should also be noted that the correlation analysis in this study was based on the combined datasets, and lack of significant correlation between some bacterial taxa and fermentation characteristics do not necessarily mean those bacterial taxa are not important. Furthermore, as revealed by Shabat et al. (2016), the functional characteristics of a small number of species can also have a large impact on community structure and ecosystem functioning. Therefore, as recommended by other researchers (Hanage, 2014; Patra and Yu, 2015), much work is still needed to confirm these correlations and to determine their causality.

## Conclusion

Nisin had greater effects than monensin in inhibiting methane production and decreasing acetate-propionate ratio. Most importantly, nisin decreased methane production without decreasing feed digestion, which is inhibited by monensin. The microbiota analysis revealed that nisin and monensin caused different alterations in the rumen microbiota and fermentation characteristics. These findings suggest that nisin can be more effective and practical than monensin in modulating rumen fermentation and mitigating methane emission, and nisin may be a potential alternative to monensin. However, future *in vivo* studies are needed to further validate nisin’s usefulness and efficacy in modifying rumen fermentation.

## Author Contributions

WZ and JS conceived and designed the experiments. JS and ZL performed the experiments. JS and ZY analyzed the data. JS wrote the paper. ZY and WZ revised the paper. All authors agree to be accountable for all aspects of the work.

## Conflict of Interest Statement

The authors declare that the research was conducted in the absence of any commercial or financial relationships that could be construed as a potential conflict of interest.

## Footnotes

1 http://drive5.com/uparse/
