# Supplementary material for: Monensin and Nisin Affect Rumen Fermentation and Microbiota Differently In Vitro
Source: Front Microbiol. 2017 Jun 16;8:1111. doi: 10.3389/fmicb.2017.01111 (PMC5472720; doi:10.3389/fmicb.2017.01111)
Supplement: Supplementary file 1 [file Table_1.docx]

Table S1. Primers used for real-time PCR quantification of rumen target organisms

| Target organisms | Primers sequences (5'→3') | Annealing temp. (°C) | Amplicon length (bp) | References |
| --- | --- | --- | --- | --- |
| Total bacteria | F: CGGCAACGAGCGCAACCC | 60 | 161 | Denman and Mcsweeney (2006) |
|  | R: CCATTGTAGCACGTGTGTAGCC |  |  |  |
| Fungi | F: GAGGAAGTAAAAGTCGTAACAAGGTTTC | 60 | 120 | Denman and Mcsweeney (2006) |
|  | R: CAAATTCACAAAGGGTAGGATGATT |  |  |  |
| Protozoa | F: GCTTTCCGWTGGTAGTGTATT | 54 | 223 | Sylvester et al. (2004) |
|  | R: CTTGCCCTCYAATCGTWCT |  |  |  |
| Methanogens  *mcr*A | F: TTCGGTGGATCDCARAGRGC | 60 | 190 | Denman et al. (2007) |
|  | R: GBARGTCGWAWCCGTAGAATCC |  |  |  |
| *C. sticklandii* | F: ATCAAAGAATTTCGGATAGG | 61 | 442 | Patra and Yu (2014) |
|  | R: CAAGTTCACCAGTTTCAGAG |  |  |  |
| *C. aminophilum* | F: ACGGAAATTACAGAAGGAAG | 57 | 560 | Patra and Yu (2014) |
|  | R: GTTTCCAAAGCAATTCCAC |  |  |  |
