# Supplementary material for: Monensin and Nisin Affect Rumen Fermentation and Microbiota Differently In Vitro
Source: Front Microbiol. 2017 Jun 16;8:1111. doi: 10.3389/fmicb.2017.01111 (PMC5472720; doi:10.3389/fmicb.2017.01111)
Supplement: Supplementary file 2 [file Table_2.DOCX]

Table S2. Effects of nisin and monensin addition on relative abundance of ruminal bacteria at operational taxonomic units (OTUs) level that each accounted for ≥ 0.5 % of total sequences in at least 1 treatment.

| OTU ID | Taxonomic lineage^*^ | Relative abundance (%) | | | | SEM | *P*-value |
| --- | --- | --- | --- | --- | --- | --- | --- |
|  |  | NC | MON | NI1 | NI5 |  |  |
| 368 | Pro; Gammaproteobacteria; Aeromonadales; Succinivibrionaceae; Ruminobacter | 11.77^b^ | 13.89^a^ | 10.22^c^ | 10.00^c^ | 0.394 | <0.01 |
| 304 | Pro; Gammaproteobacteria; Aeromonadales; Succinivibrionaceae; Ruminobacter | 9.9^b^ | 12.31^a^ | 8.70^b^ | 9.00^b^ | 0.309 | <0.01 |
| 507 | Pro; Gammaproteobacteria; Aeromonadales; Succinivibrionaceae; Ruminobacter | 7.45^a^ | 7.35^a^ | 5.58^b^ | 5.20^b^ | 0.196 | <0.01 |
| 823 | Pro; Gammaproteobacteria; Aeromonadales; Succinivibrionaceae; Ruminobacter | 1.20^b^ | 1.49^a^ | 1.10^b^ | 1.10^b^ | 0.059 | <0.01 |
| 598 | Pro; Gammaproteobacteria; Aeromonadales; Succinivibrionaceae; Succinivibrio | 5.41^c^ | 12.19^a^ | 10.20^b^ | 12.96^a^ | 0.265 | <0.01 |
| 138 | Pro; Gammaproteobacteria; Aeromonadales; Succinivibrionaceae; Succinimonas | 0.97^b^ | 1.24^a^ | 1.09^ab^ | 1.22^a^ | 0.046 | <0.01 |
| 219 | Pro; Gammaproteobacteria; Aeromonadales; Succinivibrionaceae; Succinivibrio | 0.61^b^ | 1.48^a^ | 1.18^a^ | 1.61^a^ | 0.148 | <0.01 |
| 472 | Pro; Gammaproteobacteria; Aeromonadales; Succinivibrionaceae; Unclassified Succinivibrionaceae | 0.86^c^ | 4.11^a^ | 1.23^c^ | 3.09^b^ | 0.223 | <0.01 |
| 555 | Pro; Gammaproteobacteria; Aeromonadales; Succinivibrionaceae; Unclassified Succinivibrionaceae | 0.36^b^ | 0.63^a^ | 0.54^a^ | 0.63^a^ | 0.039 | <0.01 |
| 682 | Pro; Gammaproteobacteria; Aeromonadales; Succinivibrionaceae; Unclassified Succinivibrionaceae | 0.24^c^ | 0.74^a^ | 0.31^c^ | 0.45^b^ | 0.024 | <0.01 |
| 791 | Fir; Clostridia; Clostridiales; Lachnospiraceae; Oribacterium | 2.49^c^ | 8.35^a^ | 5.06^b^ | 8.15^a^ | 0.199 | <0.01 |
| 646 | Fir; Clostridia; Clostridiales; Lachnospiraceae; Oribacterium | 0.26^c^ | 0.92^a^ | 0.67^b^ | 0.89^a^ | 0.050 | <0.01 |
| 459 | Fir; Clostridia; Clostridiales; Lachnospiraceae; Pseudobutyrivibrio | 3.05^c^ | 1.08^d^ | 5.60^a^ | 4.19^b^ | 0.116 | <0.01 |
| 806 | Fir; Clostridia; Clostridiales; Lachnospiraceae; Pseudobutyrivibrio | 0.55^b^ | 0.14^c^ | 0.72^a^ | 0.68^ab^ | 0.044 | <0.01 |
| 406 | Fir; Clostridia; Clostridiales; Lachnospiraceae; Butyrivibrio | 0.95^b^ | 0.28^d^ | 1.46^a^ | 0.77^c^ | 0.050 | <0.01 |
| 238 | Fir; Clostridia; Clostridiales; Lachnospiraceae; Anaerosporobacter | 0.71^a^ | 0.16^b^ | 0.17^b^ | 0.17^b^ | 0.021 | <0.01 |
| 409 | Fir; Clostridia; Clostridiales; Lachnospiraceae; Roseburia | 0.59^b^ | 0.20^d^ | 0.96^a^ | 0.45^c^ | 0.030 | <0.01 |
| 831 | Fir; Clostridia; Clostridiales; Lachnospiraceae; Unclassified Lachnospiraceae | 3.40^a^ | 0.76^b^ | 0.80^b^ | 0.80^b^ | 0.088 | <0.01 |
| 206 | Fir; Clostridia; Clostridiales; Lachnospiraceae; Unclassified Lachnospiraceae | 0.37^b^ | 0.08^c^ | 0.52^a^ | 0.15^c^ | 0.025 | <0.01 |
| 777 | Fir; Clostridia; Clostridiales; Lachnospiraceae; Unclassified Lachnospiraceae | 0.14^d^ | 1.28^a^ | 0.39^c^ | 1.03^b^ | 0.039 | <0.01 |
| 470 | Fir; Clostridia; Clostridiales; Christensenellaceae; Unclassified Christensenellaceae | 3.18^a^ | 0.95^b^ | 1.13^b^ | 0.98^b^ | 0.086 | <0.01 |
| 746 | Fir; Clostridia; Clostridiales; Ruminococcaceae; Ruminococcus | 1.69^a^ | 0.27^b^ | 0.26^b^ | 0.23^b^ | 0.040 | <0.01 |
| 402 | Fir; Negativicutes; Selenomonadales; Veillonellaceae; Selenomonas | 0.85^c^ | 2.69^a^ | 1.91^b^ | 2.83^a^ | 0.091 | <0.01 |
| 516 | Fir; Negativicutes; Selenomonadales; Veillonellaceae; Selenomonas | 0.19 | 0.70 | 0.41 | 0.82 | 0.028 | 0.09 |
| 840 | Fir; Negativicutes; Selenomonadales; Acidaminococcaceae; Succiniclasticum | 0.55^b^ | 1.57^a^ | 0.78^b^ | 0.86^b^ | 0.152 | <0.01 |
| 629 | Fir; Negativicutes; Selenomonadales; Veillonellaceae; Anaerovibrio | 0.12^c^ | 0.63^a^ | 0.18^c^ | 0.37^b^ | 0.025 | <0.01 |
| 500 | Fir; Bacilli; Lactobacillales; Streptococcaceae; Streptococcus | 1.61^a^ | 0.83^b^ | 0.68^c^ | 0.75^bc^ | 0.033 | <0.01 |
| 706 | Bac; Bacteroidia; Bacteroidales; Prevotellaceae; Prevotella | 1.49^a^ | 1.09^b^ | 1.53^a^ | 0.54^c^ | 0.071 | <0.01 |
| 203 | Bac; Bacteroidia; Bacteroidales; Prevotellaceae; Prevotella | 1.45^b^ | 0.29^d^ | 1.80^a^ | 0.89^c^ | 0.095 | <0.01 |
| 234 | Bac; Bacteroidia; Bacteroidales; Prevotellaceae; Prevotella | 1.00^a^ | 0.21^c^ | 0.76^b^ | 0.33^c^ | 0.044 | <0.01 |
| 435 | Bac; Bacteroidia; Bacteroidales; Prevotellaceae; Prevotella | 0.92^a^ | 0.33^c^ | 1.00^a^ | 0.61^b^ | 0.061 | <0.01 |
| 380 | Bac; Bacteroidia; Bacteroidales; Prevotellaceae; Prevotella | 0.80^b^ | 1.12^a^ | 1.02^ab^ | 0.84^b^ | 0.074 | 0.03 |
| 412 | Bac; Bacteroidia; Bacteroidales; Prevotellaceae; Prevotella | 0.57^a^ | 0.11^c^ | 0.60^a^ | 0.33^b^ | 0.033 | <0.01 |
| 566 | Bac; Bacteroidia; Bacteroidales; Prevotellaceae; Prevotella | 0.55^a^ | 0.24^c^ | 0.49^a^ | 0.37^b^ | 0.024 | <0.01 |
| 256 | Bac; Bacteroidia; Bacteroidales; Prevotellaceae; Prevotella | 0.51^a^ | 0.38^b^ | 0.55^a^ | 0.29^b^ | 0.031 | <0.01 |
| 771 | Bac; Bacteroidia; Bacteroidales; Prevotellaceae; Prevotella | 0.50^a^ | 0.22^b^ | 0.52^a^ | 0.31^b^ | 0.046 | <0.01 |
| 436 | Bac; Bacteroidia; Bacteroidales; Prevotellaceae; Prevotella | 0.45 | 0.58 | 0.61 | 0.63 | 0.046 | 0.07 |
| 632 | Bac; Bacteroidia; Bacteroidales; Rikenellaceae; Unclassified Rikenellaceae | 1.60^a^ | 0.63^c^ | 0.95^b^ | 0.47^c^ | 0.066 | <0.01 |
| 65 | Bac; Bacteroidia; Bacteroidales; Rikenellaceae; Unclassified Rikenellaceae | 0.90^b^ | 0.32^c^ | 1.08^a^ | 0.95^ab^ | 0.051 | <0.01 |
| 294 | Bac; Bacteroidia; Bacteroidales; Rikenellaceae; Unclassified Rikenellaceae | 0.64^b^ | 0.21^c^ | 0.93^a^ | 1.05^a^ | 0.049 | <0.01 |
| 431 | Bac; Bacteroidia; Bacteroidales; Rikenellaceae; Unclassified Rikenellaceae | 0.41^c^ | 0.14^d^ | 0.66^b^ | 1.03^a^ | 0.046 | <0.01 |
| 439 | Bac; Bacteroidia; Bacteroidales; Rikenellaceae; Unclassified Rikenellaceae | 0.30^b^ | 0.14^c^ | 0.26^b^ | 0.69^a^ | 0.032 | <0.01 |
| 307 | Bac; Bacteroidia; Bacteroidales; Bacteroidaceae; Bacteroides | 0.28^c^ | 0.88^a^ | 0.29^c^ | 0.55^b^ | 0.025 | <0.01 |
| 230 | Spi; Spirochaetes; Spirochaetales; Spirochaetaceae; Treponema | 0.74^a^ | 0.05^b^ | 0.12^b^ | 0.09^b^ | 0.042 | <0.01 |
| 496 | Spi; Spirochaetes; Spirochaetales; Spirochaetaceae; Treponema | 0.31^b^ | 0.05^c^ | 0.38^b^ | 0.52^a^ | 0.043 | <0.01 |
| 527 | Fib; Fibrobacteria; Fibrobacterales; Fibrobacteraceae; Fibrobacter | 0.31^b^ | 0.03^d^ | 0.60^a^ | 0.15^c^ | 0.023 | <0.01 |

NC = negative control (no additives); MON = monensin, 5 μM; NI1 = nisin, 1μM; NI5 = nisin, 5μM.

^a-d^Means within a row with different superscripts differ (*P* < 0.05).

*Pro, *Proteobacteria*; Fir, *Firmicutes*; Bac, *Bacteroidetes*; Spi, *Spirochaetae*; Fib, *Fibrobacteres*; Fus, *Fusobacteria*.

The OTU that were affected differently by monensin and nisin are highted in yellow.
